# Relationship between stereopsis and vision-related quality of life following intravitreal ranibizumab injections for central retinal vein occlusion

**DOI:** 10.1038/s41598-021-00094-z

**Published:** 2021-10-14

**Authors:** Fumiki Okamoto, Mizuki Tomioka, Tomoya Murakami, Shohei Morikawa, Yoshimi Sugiura, Takahiro Hiraoka, Tetsuro Oshika

**Affiliations:** Department of Ophthalmology, Faculty of Medicine, University of Tsukuba, 1-1-1 Tennoudai, Tsukuba, Ibaraki 305-8575 Japan

**Keywords:** Retinal diseases, Vision disorders

## Abstract

The study aimed to evaluate changes in stereopsis and vision-related quality of life (VR-QOL) in patients with central retinal vein occlusion (CRVO) following intravitreal ranibizumab injection (IVR) and investigate the relationship between stereopsis and VR-QOL. This study included 23 treatment-naïve patients with non-ischemic CRVO and 13 age-matched normal controls. Stereopsis, best-corrected visual acuity (BCVA), VR-QOL, and retinal microstructures were examined pre-treatment and 12 months post-treatment. The Titmus Stereo Test (TST) and TNO stereotest (TNO) were used to evaluate stereopsis. VR-QOL was evaluated using the 25-item National Eye Institute Visual Function Questionnaire (VFQ-25). IVR immediately and significantly improved the TST values, TNO values, composite VFQ-25 score, BCVA, and central foveal thickness in patients with CRVO. The 12-month post-treatment TST and TNO values were significantly worse in the CRVO group compared to those in the normal group. At the baseline, the composite VFQ-25 score significantly correlated only with the TST value. Multivariate analysis revealed significant associations between the 12-month post-treatment composite VFQ-25 score and the baseline and 12-month post-treatment TNO values. In conclusion, IVR immediately improved stereopsis in CRVO, albeit below normal levels. Stereopsis (not visual acuity) was associated with pre- and post-treatment VR-QOL in patients with CRVO.

## Introduction

Stereopsis, which is one of the most advanced visual functions, is the ability to perceive the depth of the field through the parallax of images formed by both eyes. Strabismus is among the typical ocular disorders in which stereopsis is compromised owing to impaired coordination between both eyes. On the other hand, stereopsis can also be impaired owing to visual dysfunction in one eye. The most common parameter of visual function that affects stereopsis is visual acuity. Previous experimental studies have shown that blurred vision in one eye affects stereopsis^1–5^. Several studies have reported the impairment of stereopsis in unilateral retinal diseases such as retinal detachment (RD)^6,7^ epiretinal membrane (ERM)^8,9^, macular hole (MH)^10,11^, and branch retinal vein occlusion (BRVO)^12^. Moreover, the stereopsis of patients with these conditions were worse than those of normal participants even following successful treatment^6,9,11,12^. However, no study has investigated stereopsis in patients with central retinal vein occlusion (CRVO).

The traditional ophthalmologic measures of clinical outcome, such as visual acuity, are increasingly being complemented by assessments of patients' visual function and perceived quality of life (QOL). The 25-item National Eye Institute Visual Function Questionnaire (VFQ-25) is a vision-related QOL (VR-QOL) instrument designed to assess patients’ perception of their visual function and QOL^13^. The VFQ-25 has been used to track the outcomes of several retinal diseases including BRVO^14,15^, ERM^9,16^, RD^17–19^, MH^20^, proliferative diabetic retinopathy (PDR)^21^, and diabetic macular edema (DME)^21^. The CRUISE^22^ and GALILEO studies^23^ used the VFQ-25 to assess the VR-QOL in patients with CRVO and investigated the changes in the QOL following treatment. The deterioration in the VR-QOL in patients with retinal diseases is attributed to the impairment of visual acuity and various factors related to visual function. Metamorphopsia has been demonstrated to be the cause of decreased VR-QOL in patients with ERM^16,21^ and MH^20^. The deterioration in contrast sensitivity has been demonstrated to be the cause of decreased VR-QOL in PDR^12^, DME^21^, after RD^17^ and vitreous floaters^24^. Moreover, another study reported that stereopsis following RD affects the patients’ VR-QOL, especially while driving^19^. The present study aimed to assess the stereopsis and VR-QOL in patients with CRVO before and after treatment, and evaluate the relationship between stereopsis and VR-QOL.

## Results

Table 1 presents the baseline characteristics of the patients with CRVO and normal controls. This study enrolled 23 patients with CRVO and 13 normal controls. The stereopsis (TST and TNO) and composite VFQ-25 scores were significantly worse in CRVO compared to normal controls. No differences were observed between the age or sex of the participants of the CRVO and normal groups. No patient with CRVO discontinued treatment during the study period. The mean number of injections during the treatment period was 5.6 ± 2.0 (range 3–8 injections). None of the patients experienced ocular treatment-emergent serious adverse events such as glaucoma, iris neovascularization, RD, vitreous hemorrhage, and endophthalmitis. None of the patients underwent cataract surgery or panretinal laser photocoagulation in the affected eye or in the other eye during the follow-up period.

**Table 1:** Baseline characteristics of patients with central retinal vein occlusion (CRVO) and normal controls.

	CRVO	Normal controls	*p* values
Age (years)	72.2 ± 11.1	68.0 ± 6.4	*p* = 0.25
Sex (men/women)	13 / 10	6 / 7	*p* = 0.55
Titmus Stereo Test (log)	3.17 ± 0.70	1.72 ± 0.19	*p* < 0 .001*
TNO stereotest (log)	3.27 ± 0.59	1.85 ± 0.23	*p* < 0 .001*
BCVA (logMAR)	0.79 ± 0.56	−0.07 ± 0.07	*p* < 0 .001*
Central foveal thickness (µm)	770 ± 319	–	–
VFQ-25 composite score (point)	62.6 ± 16.9	–	–
Duration of disease (months)	2.1 ± 2.5	–	–

* Significant differences found between two groups (Unpaired t-test).

Values are presented as the mean ± standard deviation.

*BCVA *best-corrected visual acuity, *logMAR* logarithm of the minimum angle of resolution, *VFQ-25 *25-item National Eye Institute Visual Function Questionnaire.

### Changes in stereopsis, VR-QOL, visual acuity, and CFT in the CRVO group

**Figure 1:**
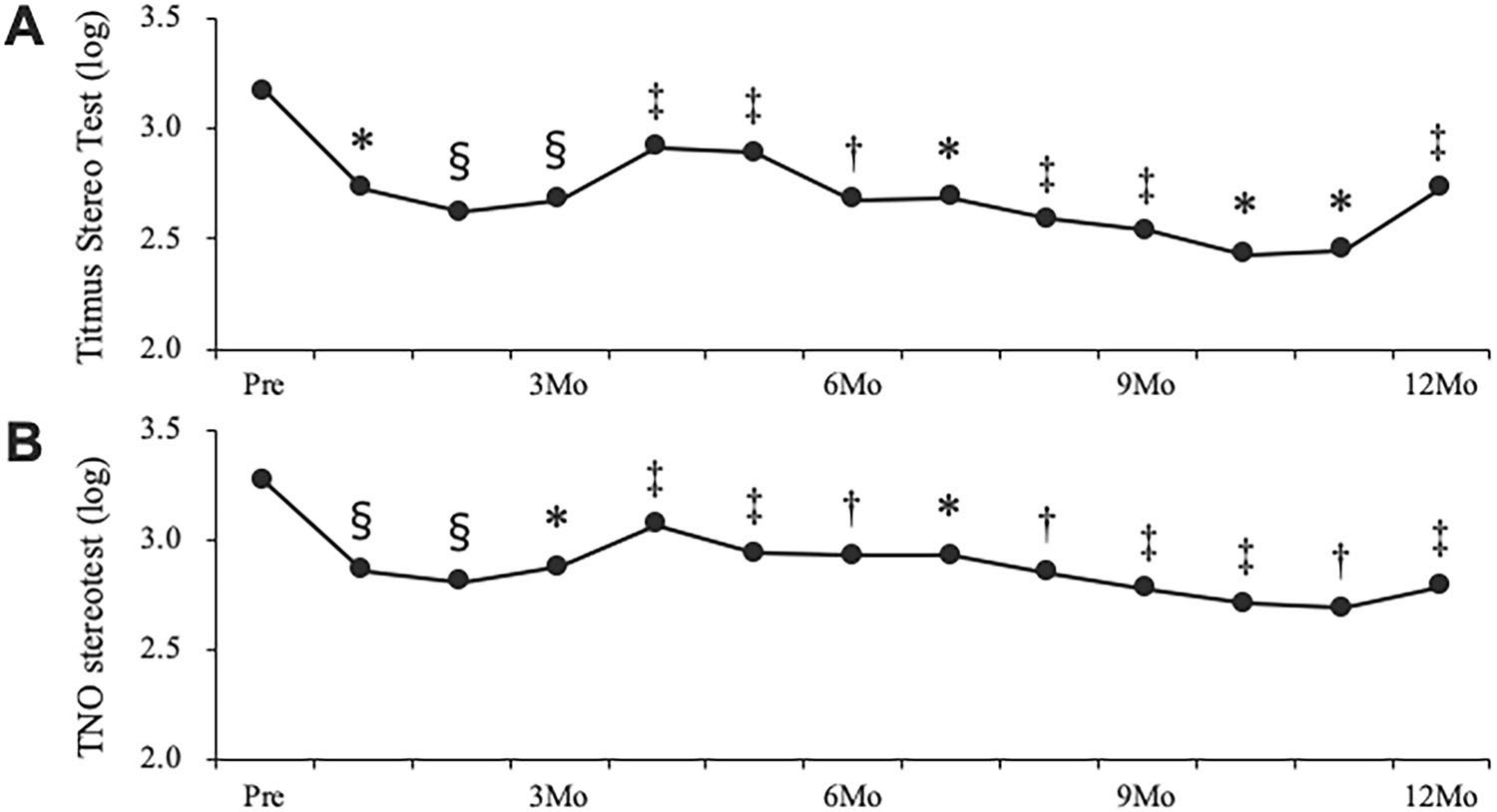
Changes in the Titmus Stereo Test (**A**) and TNO stereotest (**B**) pre- and post-treatment in patients with central retinal vein occlusion ^§^*p* < 0.001, *p < 0.005, ^†^*p* < 0.01, ^‡^*p* < 0.05 compared to baseline.

**Figure 2:**
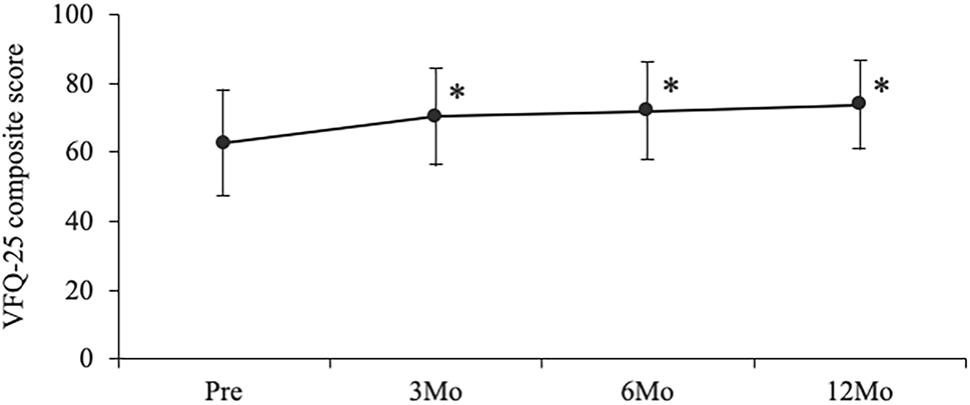
Changes in the composite VFQ-25 score pre- and post-treatment in patients with central retinal vein occlusion *p < 0.005 compared to baseline The error bars indicate standard deviations. VFQ-25 = 25-Item National Eye Institute Visual Function Questionnaire.

Figure 1 depicts changes in the stereopsis and VR-QOL. The TST values of patients with CRVO at baseline and at 3, 6, 9, and 12 months following treatment were 3.17 ± 0.70, 2.68 ± 0.84, 2.68 ± 0.79, 2.54 ± 0.77, and 2.73 ± 0.78, respectively. The TNO values of patients with CRVO at baseline and at 3, 6, 9, and 12 months following treatment were 3.27 ± 0.59, 2.88 ± 0.72, 2.93 ± 0.68, 2.78 ± 0.82, and 2.79 ± 0.76, respectively. Significant improvements were observed in the TST and TNO values at one through 12 months following IVR injection (Fig. 1A, B). The composite VFQ-25 scores at baseline and at 3, 6, and 12 months following treatment were 62.6 ± 16.9, 70.6 ± 15.6, 72.2 ± 15.8, and 74.3 ± 14.6, respectively. The composite VFQ-25 scores at 3, 6, and 12 months following treatment showed significant improvement compared to those at baseline (*p* < 0.005, *p* < 0.005, and *p* < 0.005, respectively) (Fig. 2).

**Figure 3:**
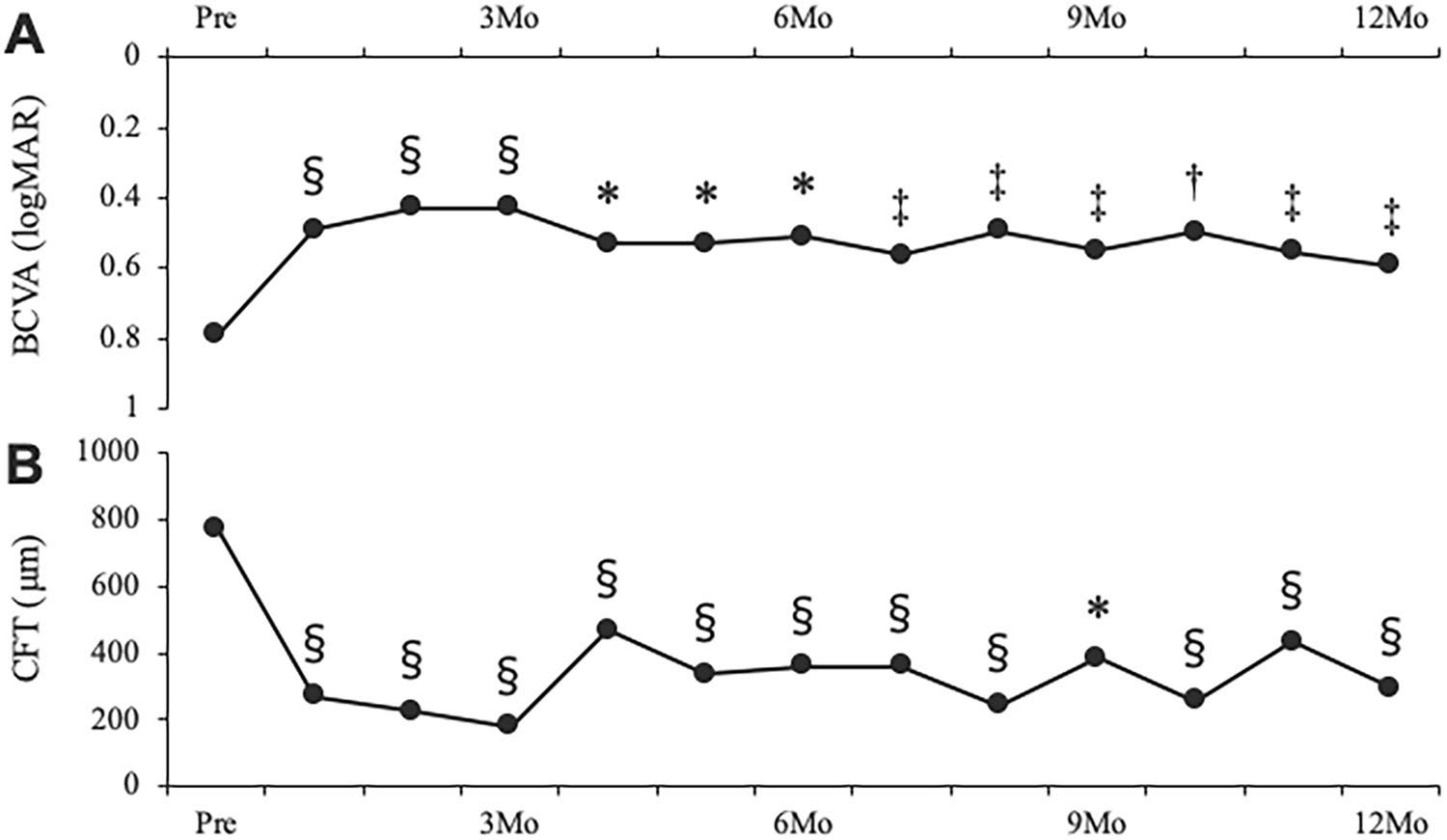
Changes in the BCVA (**A**) and CFT (**B**) pre- and post-treatment in patients with central retinal vein occlusion ^§^*p* < 0.001, *p < 0.005, ^†^*p* < 0.01, ^‡^*p* < 0.05 compared to baseline BCVA = best-corrected visual acuity, CFT = central foveal thickness.

Figure 3 depicts the changes in the BCVA and CFT. The BCVA at baseline and at 3, 6, 9, and 12 months following treatment was 0.79 ± 0.56, 0.43 ± 0.49, 0.51 ± 0.51, 0.55 ± 0.57, and 0.59 ± 0.61 logMAR, respectively. Significant improvements were observed in the BCVA at one through 12 months (Fig. 3A). The CFT at baseline and at 3, 6, 9, and 12 months following treatment was 770 ± 319, 181 ± 58, 361 ± 303, 375 ± 308, and 308 ± 259 µm, respectively. Significant improvements were also observed in the CFT at one through 12 months (Fig. 3B).

### Comparison of stereopsis between CRVO and normal controls

**Figure 4:**
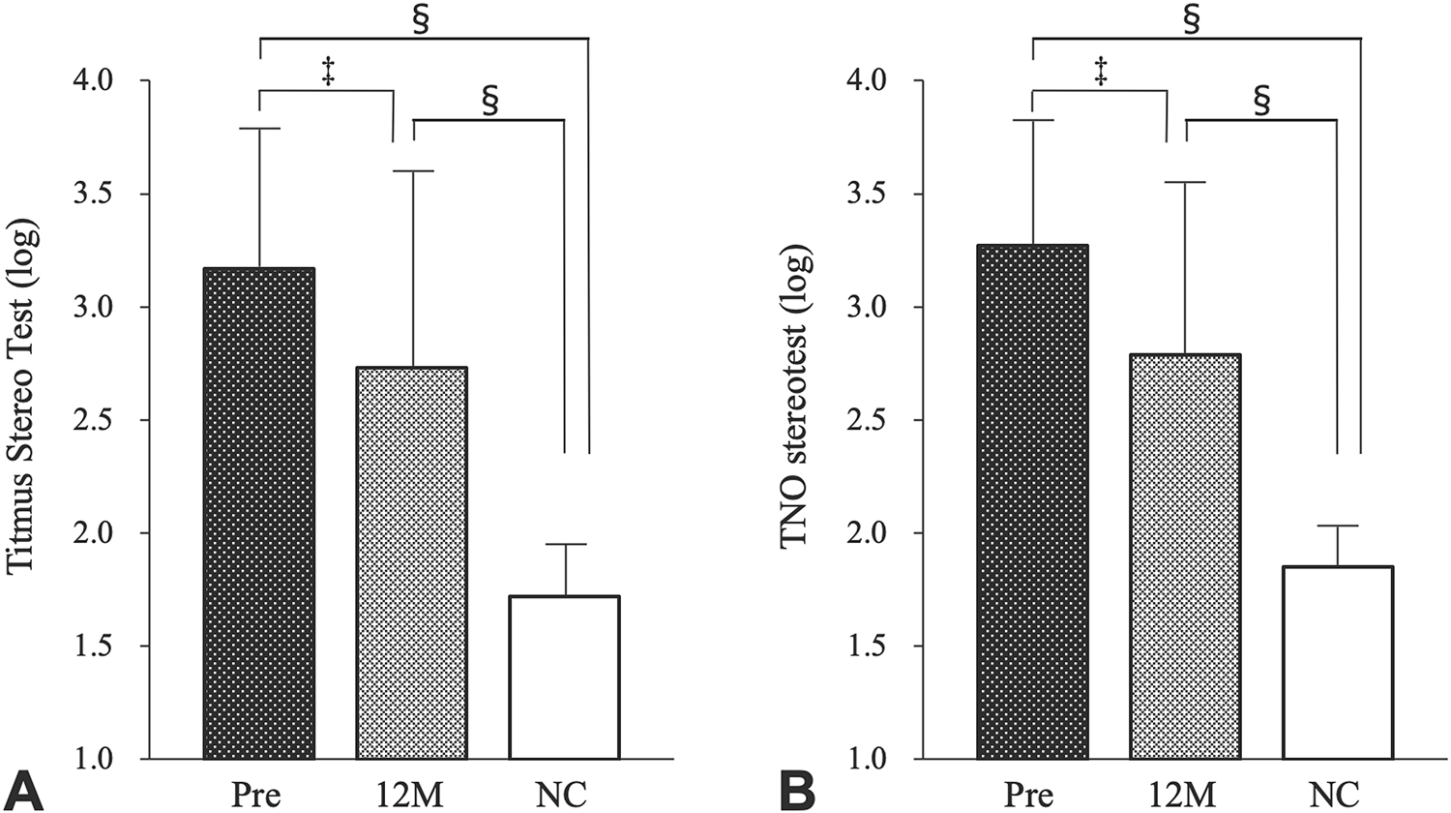
(**A**) Titmus Stereo Test values of patients with central retinal vein occlusion pre- and post-treatment 12 months and those of the normal controls (NCs) (**B**) TNO stereotest values of patients with central retinal vein occlusion pre- and post-treatment 12 months and those of the normal controls (NCs) The error bars indicate standard deviations. ^§^*p* < 0.001, ^‡^*p* < 0.05. LogMAR: logarithm of the minimum angle of resolution.

The TST and TNO values of the normal controls were 1.72 ± 0.19 and 1.85 ± 0.23, respectively. These values in CRVO before and after treatment were worse than those of the normal controls (Fig. 4A, B).

### Relationship between VR-QOL and visual functions

At the baseline, the composite VFQ-25 score significantly correlated only with the TST values, while it was not associated with the TNO and BCVA values (Table 2). At 12 months post-treatment, the composite VFQ-25 score showed a significant correlation with the TNO values and the BCVA, but not with TST (Table 2). When compared the composite VFQ-25 score 12 months post-treatment and the baseline visual functions, the TST, TNO, and BCVA values at baseline showed significant correlation with the VFQ-25 score at 12 months (Table 2). Multivariate analysis revealed that the composite VFQ-25 score obtained 12 months post-treatment was significantly associated with the TNO values at baseline (*p* = 0.011) and 12 months post-treatment (*p* = 0.019).

**Table 2:** Correlation between the composite VFQ-25 score and visual functions in central retinal vein occlusion.

	r	*P*
**Composite VFQ-25 score at baseline**		
Titmus Stereo Test at baseline	−0.450	0.036*
TNO stereotest at baseline	−0.419	0.052
BCVA at baseline	−0.184	0.412
**Composite VFQ-25 score 12 months post-treatment**		
Titmus Stereo Test at 12 months post-treatment	−0.419	0.094
TNO stereotest at 12 months post-treatment	−0.660	0.004*
BCVA at 12 months post-treatment	−0.554	0.021*
**Composite VFQ-25 score 12 months post-treatment**		
Titmus Stereo Test at baseline	−0.482	0.043*
TNO stereotest at baseline	−0.595	0.009*
BCVA at baseline	−0.553	0.017*

* Significant correlations found between the parameters (Spearman rank-correlation test).

*BCVA* best-corrected visual acuity, *VFQ-25* 25-item National Eye Institute Visual Function Questionnaire.

### Relationship between Stereopsis and visual acuity

At the baseline, the BCVA significantly correlated with the TST values (r = 0.807, *p* < 0.001) and TNO values (r = 0.474, *p* < 0.022). At 12 months post-treatment, the BCVA significantly correlated with the TST values (r = 0.904, *p* < 0.001) and TNO values (r = 0.825, *p* < 0.022).

## Discussion

This study found that patients with CRVO had impaired stereopsis, which was worse than that in the normal controls. The baseline stereopsis (log) in the patients with CRVO in this study was 3.17 as measured by the TST and 3.27 as measured by the TNO. This study was the first to investigate stereopsis in patients with CRVO. The impairment in the visual function of one eye is known to compromise stereopsis. Prior studies have investigated the disturbance of stereopsis in patients with unilateral retinal diseases, including RD^6,7^, ERM^8,9^, MH^10,11^, and BRVO^12^. The stereopsis in patients with ERM was 2.35 on the TST and 2.84 on the TNO^9^, 2.7 on the TST and 2.8 on the TNO in patients with MH^11^, and 2.72 on the TST and 2.72 on the TNO in patients with BRVO^12^. IVR immediately improved stereopsis in patients with CRVO; however, it could not restore it to the level of normal controls. The stereopsis was 2.73 on the TST and 2.79 on the TNO 12 months post-treatment in patients with CRVO in this study. The stereopsis following treatment for ERM was 2.19 on the TST and 2.63 on the TNO^9^, 2.2 on the TST and 2.4 on the TNO in MH^11^, and 2.06 on the TST and 2.32 on the TNO in BRVO^12^. Therefore, the degree of stereopsis impairment in CRVO is worse than that in other retinal diseases both before and after treatment.

The BCVA and CFT improved immediately following treatment in patients with CRVO and remained stable throughout the subsequent 12-month period. The 12-month improvement in the BCVA was 0.2 logMAR (10 letters) in this study. The 12-month BCVA following treatment was 13.9 and 16.9 letters better than that of the baseline in the CRUISE^22^ and GALILEO studies^23^, respectively. The reason for the comparatively lower improvement in the BCVA in our study than that in the other studies could be attributed to the inclusion criteria. The BCVA for eligibility in the CRUISE^22^ and GALILEO studies^23^ were 20/40 to 20/320. On the other hand, our study did not set any BCVA value as an eligibility criterion, and thus, the BCVA in our study population ranged from 20/20 to 20/2000. The improvement in visual acuity could be comparatively lesser than that in the previous studies because our study included patients with extremely poor vision and good vision.

The composite VFQ-25 score improved from 62.6 points before treatment to 74.3 points 12 months following treatment. The mean increase in the composite VFQ-25 score (from baseline) was 9.6 points at 6 months, and 11.7 points at 12 months following treatment in this study. The mean increase from the baseline VFQ-25 composite score was 7.1 points every 6 months following treatment, and 7.8 and 7.1 points every 12 months following treatment, respectively, in the GALILEO^23^ and CRUISE studies^22^. On the other hand, the mean number of injections during the 12-month period in our study was 5.6, which was lower than that in the CRUISE (9.8) and GALILEO (11.8) studies. Despite these results, the composite VFQ-25 score in our study improved as much or more than that in other studies, suggesting that 3 + PRN is acceptable as the standard treatment for CRVO from the perspective of the QOL. However, there are problems in comparing VFQ-25 values in different study settings and among different retinal diseases. In the country and race of the institution where the study was performed, retinal diseases including CRVO have their own characteristics, and it is difficult to compare them unitarily.

The baseline composite VFQ-25 score was associated with the TST values, and not the BCVA and TNO. The composite VFQ-25 score12 months post-treatment was not related to the TST, but to the TNO values and BCVA 12 months post-treatment. Moreover, multiple regression analysis determined the TNO value as a prognostic factor for the VR-QOL in patients with CRVO. The results of the two types of stereo tests that affect VR-QOL were inconsistent in this study. This discrepancy may be attributed to the different index sizes used in the two stereo tests. The stimulus used for assessing fine stereopsis in TNO was much larger than that used in the TST circles. The TST circles subtend a visual angle of 0.7°, while those in TNO subtend an angle of 8.5°. The diameters of the fovea and foveola were 1500 and 350 μm, respectively, with visual angles of approximately 5° and 1.2°, respectively. In CRVO patients with CME, visual function is expected to be impaired in a large area, including the central fovea. At baseline, the CFT in particular is very thick and visual function is poor (770 μm in this study). Therefore, TST, which targets a small area near the central fossa, may have been associated with VR-QOL. After 1 year of treatment, the CFT becomes thinner (308 μm in this study), so the visual impairment of not only the foveal area but also the entire posterior pole becomes apparent, and TNO, which targets a large area, may have been associated with VR-QOL. The relationship between visual acuity and VR-QOL is well known in diseases such as diabetic retinopathy^25^ and uveitis^26^. In addition, the relationship between visual functions other than visual acuity and VR-QOL has been reported in several retinal diseases. Previous studies reported that VR-QOL was affected by metamorphopsia in patients with ERM^16,21^ and MH^20^ and that the VR-QOL was associated with contrast sensitivity in PDR^21^ DME^21^, after RD^17^, and vitreous floaters^24^. Moreover, stereopsis was associated with the VR-QOL, especially during driving, following RD surgery^19^. Our results suggest that stereopsis is an important factor affecting the QOL of patients with CRVO.

The BCVA significantly correlated with TST and TNO at baseline and at 12 months post-treatment in this study. The most common parameter of visual function that affects stereopsis is visual acuity. Previous experimental studies have shown that poor visual acuity in one eye impairs stereopsis^1–5^. Decreased visual acuity was also associated with impaired stereopsis in unilateral retinal diseases such as RD, ERM, MH and BRVO^6–12^. Therefore, these results in CRVO patients are consistent with previous reports. However, impaired stereopsis, not decreased visual acuity, affected VR-QOL in this study. This may mean that activities of daily living affect binocular visual function more than visual acuity.

The limitations of this study include its small sample size and short follow-up duration. We evaluated the patients for 12 months following treatment. In the COPERNICUS study, the BCVA 24 months following treatment with intravitreal aflibercept injection was 3.2 letters lower than that following 12 months in patients with CRVO^27^. Since CRVO is a retinal disease with a poor prognosis, long-term monitoring of the visual functions and QOL is important. Other factors known to affect stereopsis include pupil size^28^, accommodation^5^, and eye dominance^29^; however, these factors were not assessed in this study. Nevertheless, the influence of pupil size and eye dominance on stereopsis is negligible, and is unlikely to have affected our results. Even if the pupil size changed from 1 to 6 mm, the change in TST score was 0.18 in a previous study^28^, while the TST score changed by 0.2 with the change in the dominant eye^29^. Future studies with larger sample sizes and longer follow-up periods incorporating other factors are needed to further our understanding of stereopsis and visual function factors in patients with CRVO.

## Methods

### Study design

This multicenter, open-label, prospective study was conducted in accordance with the Declaration of Helsinki and with the approval of the Institutional Review Board of the University of Tsukuba Hospital and Mito Kyodo General Hospital. All the patients and normal subjects provided informed consent prior to inclusion in the study. Treatment-naïve patients with non-ischemic CRVO who were referred to the Tsukuba University Hospital or Mito Kyodo General Hospital were enrolled in this study. The inclusion criteria for participation were as follows: (1) center-involving macular edema secondary to CRVO, (2) central foveal thickness (CFT) > 250 μm, (3) patients aged 18 years or above, but younger than 85 years, and (4) patients who provided written informed consent. The exclusion criteria were as follows: (1) previous history of ophthalmic disorders in affected and contralateral eyes, except mild refractive errors and mild cataract, (2) pseudophakia in only one eye, (3) patients who underwent treatment for macular edema within the last 90 days (including sub-tenon triamcinolone acetonide, intravitreal bevacizumab, intravitreal ranibizumab [IVR], intravitreal aflibercept, topical steroid, and carbonic anhydrase inhibitors), (4) patients who underwent intraocular surgery within the past 90 days, (5) patients with poorly controlled hypertension and diabetic mellitus, and (6) patients who underwent laser treatment within the last 30 days. We also included age-matched normal controls in this study.

### Study visits and assessments

The Titmus Stereo Test (TST) and the TNO stereotest (TNO), which are tests of stereopsis, best-corrected visual acuity (BCVA), and retinal microstructure were examined every month before treatment and over a period of 12 months after treatment. The VR-QOL was examined before treatment and at 3, 6, and 12 months following treatment. We converted the BCVA measured using the Landolt chart to the logarithm of the minimum angle of resolution (logMAR) for use in the subsequent analysis. Because the retinal damage in CRVO is not limited to the foveal area but covers a wide area, we performed two stereoscopic tests with different sizes of indices. TST and the TNO were performed under appropriate spectacle corrections with a standard viewing distance of 40 cm to evaluate the stereopsis. We flipped the stereo target and asked the patient if the target was in front or behind the page, and checked the response to ensure that the patient did not use monocular clues during the TST. The results for TST and TNO were expressed in "seconds of arc." These values were converted to logarithms for statistical evaluation^11^.

The macular structure was evaluated using spectral-domain optical coherence tomography (OCT) (Cirrus high-definition OCT; Carl Zeiss, Dublin, CA). Five-line Raster Cross scanning was performed using the Cirrus analysis software version 3.0., and scans with a signal strength of more than 6/10 were considered appropriate. The CFT was evaluated using the OCT image.

The VFQ-25 was administered to investigate the VR-QOL in patients with CRVO. The VFQ-25 consists of 25 items that permit patients to self-assess specific visual symptoms and difficulty with daily activities. The responses to the 25 questions are assigned to one of the following 12 subscales: general health, general vision, ocular pain, near activities, distance activities, social functioning, mental health, role limitations, dependency, driving, color vision, and peripheral vision. The composite VFQ-25 score is calculated as the average of the 11 subscale scores, excluding “general health.” Subscales are scored on a scale of 0–100 points, with 100 indicating the best possible functioning or minimal subjective impairment. This study utilized the Japanese version of the VFQ-25, which was modified to fit Japanese culture and lifestyle. The reliability and validity of the modified NEI VFQ-25 questionnaire have been assessed, and the questionnaire was proven to accurately measure the VR-QOL in Japanese individuals^30^.

### Intraocular injections

The patients with CRVO were administered three successive monthly injections of IVR (3 injections during months 0–2) (0.5 mg. Lucentis; Genetech) followed by *pro re nata* administration (3 + PRN). After 3 injections (month 2), the participants were examined monthly and treated with intravitreal injections on a PRN basis according to the retreatment protocol. The criteria for PRN re-injection were as follows: (1) CFT ≥ 300 μm in the study eye as assessed by OCT, (2) detection of new cystoid changes in the retina, retinal bleeding or subretinal fluid on OCT, and (3) a decrease in visual acuity > 0.1 logMAR compared to the values obtained at the last visit.

The injection protocol was as follows: an eyelid opener was applied following topical anesthesia instillation and the injection site was washed with povidone iodine. A 30-gauge needle was inserted through the pars plana and 0.05 mL of ranibizumab was injected. All the procedures were performed at our clinic by experienced vitreoretinal surgeons (T.M., Y.S., and S.M.).

### Statistical analysis

The mean and standard deviation values were calculated for each parameter. The unpaired t-test was used to compare the age, stereopsis, and BCVA between patients with CRVO and normal controls. The chi-squared test was performed to determine the presence of any sex-based between-group differences. The Wilcoxon signed-rank test was performed to investigate changes in the visual function (TST, TNO, and BCVA), CFT, and composite VFQ-25 score. The associations between stereopsis and the composite VFQ-25 score and BCVA were examined using the Spearman rank-correlation test. Multivariate analysis was performed to investigate the relationship between the VR-QOL and visual functions. All the analyses were conducted using SPSS (version 27, IBC Corp., Chicago, IL, USA). *P*-values less than 0.05 were considered statistically significant.
